# Primary Cilia: A Cellular Regulator of Articular Cartilage Degeneration

**DOI:** 10.1155/2022/2560441

**Published:** 2022-09-23

**Authors:** Haiqi Zhou, Sha Wu, Huixian Ling, Changjie Zhang, Ying Kong

**Affiliations:** Department of Rehabilitation, The Second Xiangya Hospital, Central South University, Changsha 410000, China

## Abstract

Osteoarthritis (OA) is the most common joint disease that can cause pain and disability in adults. The main pathological characteristic of OA is cartilage degeneration, which is caused by chondrocyte apoptosis, cartilage matrix degradation, and inflammatory factor destruction. The current treatment for patients with OA focuses on delaying its progression, such as oral anti-inflammatory analgesics or injection of sodium gluconate into the joint cavity. Primary cilia are an important structure involved in cellular signal transduction. Thus, they are very sensitive to mechanical and physicochemical stimuli. It is reported that the primary cilia may play an important role in the development of OA. Here, we review the correlation between the morphology (location, length, incidence, and orientation) of chondrocyte primary cilia and OA and summarize the relevant signaling pathways in chondrocytes that could regulate the OA process through primary cilia, including Hedgehog, Wnt, and inflammation-related signaling pathways. These data provide new ideas for OA treatment.

## 1. Introduction

Osteoarthritis (OA) is a degenerative joint disease that severely influences the quality of life and is characterized by joint pain, swelling, stiffness, and motor dysfunction [1]. According to the Global Burden of Disease Study 2019, OA affects 7% of the global population, more than 500 million people worldwide, which is the 15th highest cause of years lived with disability (YLDs) worldwide and is responsible for 2% of the total global YLDs [2]. Its pathological features are dominated by cartilage degeneration associated with subchondral bone remodeling, osteophyte formation, and synovial inflammation, involving complex inflammatory, mechanical, and metabolic factors [3, 4]. However, the exact mechanism of the pathogenesis of OA is still poorly understood.

Chondrocyte, the sole cell type in articular cartilage, are essential to maintain the normal structure and integrity of cartilage matrix [5]. Nevertheless, due to its complex anisotropic structure and avascularity, the cartilage exhibits limited self-repairing capacity. Thus, chondrocytes may cause OA-like chondrogenic degeneration after apoptosis. Under normal physiological conditions, chondrocyte apoptosis is important to maintain the homeostasis of various tissues in the human body, as well as to regulate normal cartilage development, while excessive apoptosis in chondrocytes causes damage to the repair and reconstruction of articular cartilage, leading to further cartilage degeneration and aggravating OA progression [6].

The primary cilium is a singular, immobile organelle present in most cells. As early as 1967, it has been demonstrated that primary cilia also present on chondrocytes [7]. Primary cilia are considered to function as cellular sensory organelles that can respond to diverse mechanical, chemical, and biological stimuli and play a key regulatory role in multiple biological processes, including cell proliferation, maturation, differentiation, and secretion. Many receptors for a variety of important signaling pathways are expressed in the primary cilia, such as Hedgehog (Hh), Wnt, Notch, transforming growth factor *β* (TGF-*β*), G protein-coupled receptor, and calcium signaling pathways. Dysfunctions or genetic mutations of the primary cilium cause a number of human diseases, classified as ciliopathies [8], such as polycystic kidney diseases [9], retinitis pigmentosa [10], and Bardet–Biedl syndrome [11], which can also manifest as abnormal cartilage formation [12].

Currently, several reports have associated the pathogenesis of OA with the function of primary cilia. Although the underlying mechanisms have not been clearly defined, there is growing evidence that primary cilia play an important role in cartilage degeneration in OA. Therefore, this paper will discuss the following aspects: the basic structure and material transport of primary cilia, the morphology of chondrocyte primary cilia, and their related signaling regulation mechanisms. We hope that this literature review will provide a new idea for OA treatment.

## 2. The Structure of Primary Cilia

**Figure 1:**
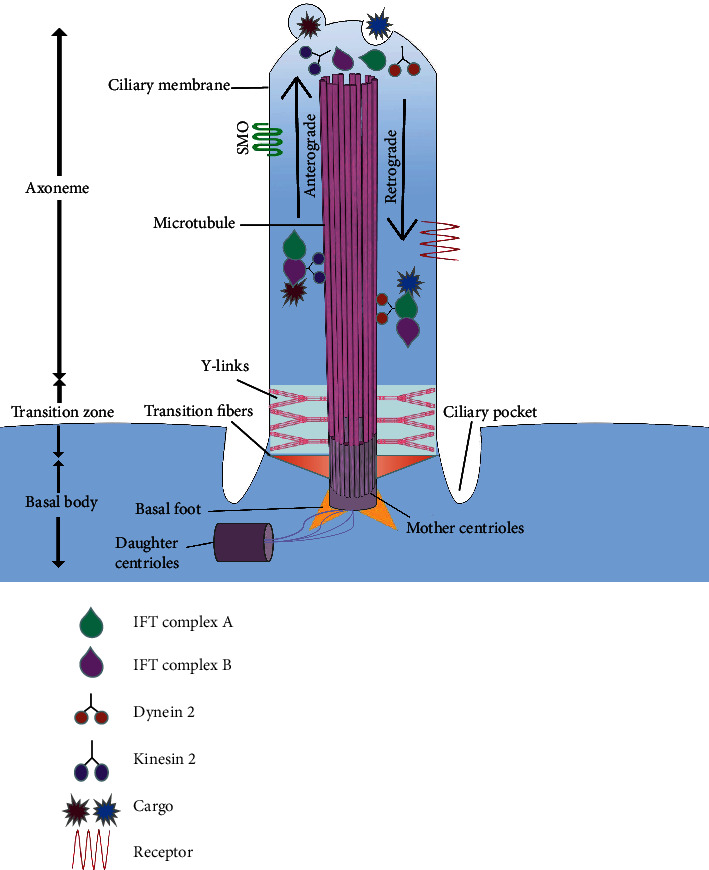
The structure of primary cilium. The cilium structure mostly contains three parts: the basal body, transition zone, and axoneme. The basal body locates at the ciliary bottom, which is associated with other cellular organelles (such as the nucleus and Golgi body). The transition zone is between the basal body and the axoneme, acting as a “gatekeeper”; it controls the entry and exit of various substances and proteins. The axoneme consists of nine pairs of doublet microtubules and covers with a ciliary membrane, which is the main component of primary cilia sensing the extracellular signal.

Primary cilia are a highly specialized cell structure that protrudes from the surface of the cell membrane [13]. Each cell has only one cilium, which is widely present in most types of tissues. As a cellular “antenna,” it receives various signals from the extracellular environment, including light, low molecular weight chemicals, proteins, and mechanical stimuli [14]. The structure of the cilium mainly contains three parts: the basal body, the transition zone, and the axoneme (Figure 1). The basal body is derived from the mother centriole, containing ciliary rootlets (or striated rootlets), which is providing structural support for primary cilia [15]. In cycling cells, the centrosome is made up of mother centrioles, daughter centrioles, and a protein matrix called pericentriolar material. After mitosis, the paired centrioles become disengaged. In G0 cell cycle stage, the mother centriole is associated with Golgi-derived ciliated vesicles and migrates near the plasma membrane to become a basal body [15, 16]. Subsequently, the distal appendages carried by the mother centriole form transition fibers (connected to the cell membrane), while the subdistal appendages become the basal foot [17, 18]. The transition zone, an intermediate region between the basal body and the axoneme, consists of transition fibers and Y-links, which act as a ciliary gate, controlling soluble proteins and selected components in and out of cilia [19–21]. Here, the nine microtubules of the basal body are transformed from triplets into doublets, which form the backbone of the ciliary axoneme (known as the 9 + 0 arrangement, it lacks one pair of central microtubules and dynein arms, which is different from motor cilia) [22]. In addition, the axoneme is covered with a ciliary membrane that extends to the plasma membrane, but has different components. The ciliary membrane contains a pore complex for size-based exclusion—only specific proteins can be located in the primary cilia [23, 24]. This feature makes primary cilia form a special subcellular microdomain to play its unique signaling role. Hundreds of ciliary proteins have been identified [25, 26], and we listed some of the proteins associated with cartilage (Table 1). Remarkably, the deep hole between cell membrane and ciliary membrane is called the ciliary pocket, which engages in endocytic activity and interacts with the actin-based cytoskeleton [27].

## 3. The Material Transport of Primary Cilia

Intraflagellar transport (IFT) is a two-way cargo transport system, and all signaling within the primary cilia must be IFT-dependent to complete. Generally, it is divided into anterograde IFT (moves from the base of a cilium to the tip) and retrograde IFT (moves from the tip back to the base), which are mediated by heterotrimeric kinesin-2 and cytoplasmic dynein 2 motor (some call cytoplasmic dynein 1b motor), respectively [36, 37]. In this IFT train, when the cargo needs to be transported in the basal body, the IFT-B complex aggregates and activates kinesin-2 and moves the cargo to the tip of axoneme and they dissociate, releasing the substance; then, the new cargo comes in and kinesin-2 deactivates whereas dynein 2 activates in bonding with the IFT-A complex and transports the new cargo to the basal body [38]. This transport mechanism is essential for the structure and function of primary cilia, as targeting interference with IFT proteins in animal models leads to the loss of primary cilia and phenotypes similar to human genetic ciliopathies [39, 40]. For example, in a Col2*α*1; IFT80f/f mouse model, the deletion of IFT80 results in growth retardation, shortened growth plate, thickening of the articular cartilage, and defects of the primary cilia [41].

## 4. Chondrocyte Primary Cilia

Chondrocytes are the only cell type located in the articular cartilage. Similar to most mammalian cells, chondrocytes contain primary cilia [42]. The characteristics of primary cilia, such as location, morphology, and incidence, are usually determined by distinct differentiated cell states [43]. For example, the length of the primary cilia increased after chondrogenic induction in ATDC5 cells (one type of prechondrocyte cell line), while the incidence was apparently not altered and ranged from 80% to 88%. In recent years, many scholars have detected the morphology of primary cilia in chondrocytes. Thereinto, the vast majority of literature reports describe the location of the primary cilia, which extends from the inner chondrocyte and interacts with the extracellular matrix [44, 45]. Here are some more specific comments describing the location of the primary cilia in chondrocytes. Reportedly, in agreement with the transmission electron microscopy, the immunocytochemistry images in situ and live cell confocal ex vivo depict that the chondrocyte primary cilia from femora in mature female Balb/C mice and horses were positioned within membranous invaginations into the cell (as already mentioned above “ciliary pocket”) and close to the nucleus without protrusion in the extracellular matrix, rather than projecting out from the cell membrane at the cell margin as seen in other cell types [46, 47]. In another way, McGlashan et al. [48] reported that primary cilia in the chondrocyte/agarose constructs were lying against the chondrocyte cell membrane or extending into the extracellular microenvironment. The inconsistency of the two results may be related to the different culture methods (in vivo or in vitro, explants, agarose constructs, or cell culture) or different types and sites of cultured animal cells, suggesting that the morphological manifestations of the chondrocyte primary cilia are affected by many factors.

The orientation of primary cilia appears to be important in the development of motor and sensory functions [49]. Stockwell [50] found that the adjacent chondrocytes of sheep could not show any consistent cilia localization. Furthermore, the orientation of chondrocyte primary cilia from load bearing and nonload regions of the equine femoral condyles in the coronal plane was random, and the orientation in the superficial area and in the radiation area was different [47]. The axoneme orientation of the chondrocyte primary cilia in the superficial region is parallel to the articular surface, while others in the radiation region point directly to the articular surface. Besides, ultrastructural studies have shown that chondrocyte primary cilia are located on the basal side of individual chondrocytes in the superficial and middle cartilage zones, on either of the two sides perpendicular to the articular surface in the cartilage/bone interface region and on the side adjacent to the second cell within a chondron [46]. This seems to be somewhat different from previous findings, possibly resulting from factors such as the orientation and location of the slice.

Like previously reported mesenchymal stem cells [51], epithelial cells [52], and trophoblastic cells [53], the incidence of articular chondrocyte cilia is also a cilia per cell [49, 54]. In particular, studies have shown that the incidence of primary cilia is positively related to cell density, cell density increases, and the expression of primary cilia increases [55]. In addition, the incidence and length of primary cilia in normal chondrocytes changed by the zonal region, all of which increased with the distance from the surface of the joint [56]. At the same time, they can also be affected by the surrounding environment of chondrocytes, such as inflammation, osmotic pressure, and mechanical load. In this process, the length, incidence, and orientation of primary cilia change accordingly. The length and incidence of chondrocyte primary cilia in OA have been reported to increase [56]. Under imaging of the femoral condyle in mice, chondrocyte primary cilia length decreased significantly after hypo- or hyperosmotic stimulation while primary cilia incidence remained unchanged [46]. This indicates that primary cilia are sensitive to physical and chemical stimuli and can help chondrocytes perceive and respond to mechanical stress [29]. During prolonged periods (>24 hours) of compressive strain, primary cilia length and incidence decrease on chondrocytes from bovine metacarpophalangeal joints seeded in agarose, but the changes were reversible after removal of compression and culture for an additional 72 h [48]. Similarly, Xiang et al. [57] found that the primary cilia expression was inhibited in rat knee chondrocytes under high-intensity pressure (4000 *μ*strain) and that the length was shortened. The above findings indicate that mechanical stress is quite closely related to the chondrocyte primary cilia. We hypothesized that chondrocyte primary cilia affect cartilage development through multiple signaling pathways and influence the development of OA.

## 5. Related Signaling Regulation Mechanisms of the Chondrocyte Primary Cilium and OA

In the human articular cartilage, no new chondrocytes are produced once bone growth is completed. Therefore, damage to or apoptosis of chondrocytes will lead to an imbalance in cartilage homeostasis, the degradation of the extracellular matrix, and the degeneration of cartilage [58]. OA is a degenerative joint disease characterized by an imbalance between the catabolism and anabolism of the cartilage matrix. In this process, the primary cilia, a signal receiver, are important components of chondrocyte signal transduction, with the cilium believed to be involved in and mediating extrachondrogenic matrix degradation [59]. Mechanical stimulation is an important stimulus most commonly felt by chondrocytes and an important influencing factor in cartilage degeneration. The prevailing theory is that mechanical stimulation causes chondrocyte primary cilia to alter morphology to initiate a downstream signaling cascade [60]. In the following, we will discuss the signaling transduction pathways related to OA that have been shown to be coordinated by primary cilia, such as the Hedgehog (Hh) signal pathways and Wnt signal pathways.

### 5.1. Hedgehog Signaling Pathways

In mammals, the Hedgehog (Hh) signaling pathway is mainly composed of Hh ligands, receptors, smoothened protein (SMO), suppressor of fused (Sufu), and Gli. There are three Hh ligands: sonic hedgehog (Shh), Indian hedgehog (Ihh), and desert hedgehog (Dhh). The transmembrane receptor patched (Ptc), as a common receptor in the Hh ligand, plays a key role in the reception and transduction of Hh signals [61]. SMO, a transmembrane protein of the GPCR superfamily that acts downstream of Ptc, is an important positive regulator of Hh signaling and whose activity is regulated by Ptc [62]. Suppressor of fused (Sufu) is a highly conserved protein with strong inhibitory activity, and its removal leads to ectopic activation of the Hh pathway [63]. In the absence of Hh signaling, the interaction between the Sufu and Gli proteins plays a key role in promoting the processing of Gli into truncated repressor forms [64, 65]. As a transcription factor, Gli is a key effector of Hh activity. Among them, Gli1, which lacks the repressor domain of the N-terminal region, acts only as a transcriptional activator [66]. Gli2 and Gli3 have dual activity, but the main function of Gli3 in the Hh signaling pathway is an inhibitor (GliR), and GLI2 is an activator (GliA) [67]. Without Hh ligand, Ptc inhibits SMO activity, puts it in a closed state, increases PKA activity, promotes the phosphorylation of the Sufu and Gli complexes, processes it into a truncated repressor form of GliR at the C-terminus, and suppresses Hh target genes in the nucleus. When the Hh pathway is activated, Hh ligands bind to the Ptc receptor to release inhibition of SMO, which inhibits Gli processing, prompting the full-length Gli transcription factor into the nucleus to activate relevant target genes.

As research progresses, the role of Hh signaling in OA chondrocytes has been preliminarily clarified. Hh signaling regulates the growth and differentiation of normal chondrocytes, in which Indian hedgehog (Ihh) is the major ligand expressed in the OA cartilage [68–70]. In this regard, primary cilia are necessary for both ligand Indian hedgehog- (Ihh-) mediated and mechanical loading-mediated Hh signaling activation in chondrocytes [71, 72]. Chondrocyte hypertrophy is a common manifestation of OA. The Ihh is secreted by prehypertrophic chondrocytes to regulate hypertrophy and mineralization of chondrocytes during chondrogenesis [73]. Instead, parathyroid hormone-related protein (PTHrP) can inhibit chondrocyte hypertrophy and maintain chondrocyte proliferation [74, 75]. Multiple studies have demonstrated that increased Ihh signaling activates the PTHrP signaling pathway in hypertrophic chondrocytes, causing overexpression of genes associated with cartilage degeneration and the development of OA [76–78]. The overexpression of PTHrP also causes negative feedback, resulting in inhibition of the expression of the Ihh signal, which reverses the chondrocyte hypertrophy and protects the cartilage. The imbalance or disorder of the Ihh-PTHrP signaling pathway may be one of the mechanisms that causes cartilage degeneration in OA [79–81]. Regardless, the importance of the Ihh-PTHrP negative feedback loop is recognized in OA progression. As described above, this suggests the possibility that blocking Ihh signaling can be used as a treatment to prevent or delay cartilage degeneration. Nevertheless, Ihh knockout may not be a treatment option as it has greater uncertainty in animal experiments. The col2*α*1-Cre; Ihh^d^/Ihh^d^ mutants died shortly after birth and were smaller in size, exhibiting malformed and retarded growth of limbs with severe skeletal deformities [82]. Another Col2a1-CreER (T2); Ihh (fl/fl) mouse genetic study provides direct evidence that conditional knockout of Ihh in chondrocytes can reduce catabolism, increase anabolic biomarkers production, and slow OA progression [83]. These findings suggest that Ihh is possibly a target point in OA treatment.

**Figure 2:**
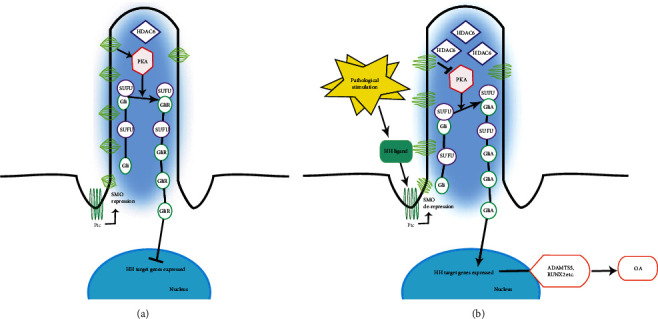
A brief diagram of Hh signaling under pathological stimuli. Histone deacetylase 6 (HDAC6) is a tubulin deacetylase that regulates the length of the primary cilia. Under normal conditions, the expression of HDAC6 maintains the length of the primary cilia. When the Hh ligand is not activated, Ptc inhibits SMO activity, puts it in a closed state, increases PKA activity, promotes phosphorylation of the SuFu and Gli complex, processes into a truncated repressor form of Gli repressor (GliR), and suppresses Hh target genes in the nucleus (a). Upon pathological stimulation, HDAC6 expression is abnormally increased, promoting primary ciliary elongation and activating the Hh signaling pathway. When the Hh ligand binds to the Ptc receptor to release inhibition of SMO, Smo inhibits Gli processing, causing the full-length Gli activator (GliA) to enter the nucleus to activate relevant target genes such as ADAMTS5 and RUNX2, which causes changes in OA.

The primary cilia are the signal transduction centers of Hh [84], and their length is thought to regulate the function of the cilium, partially controlled by IFT activity. In the articular cartilage, the primary cilia length of OA increases, and Hh signaling is activated. Lin et al. and Thompson et al. [85, 86] demonstrated that the activation level of the Hh signal in human and animal samples is positively correlated with the severity of OA. Upregulation of the Hh signal leads to an increase in osteoarthritis markers, such as ADAMTS5, RUNX2, and MMP13, which accelerate the progression of OA. The pathological mechanism may be that the increased cilia length and incidence of articular cartilage are affected by pathological stimulation, resulting in abnormal activation of Hh signals and promoting the cartilage degeneration in OA (Figure 2). Therefore, blocking Hh signals may be a new treatment for osteoarthritis. Moreover, it has been reported that lithium chloride injection in animal models of OA can protect joints and reduce cartilage damage by regulating primary cilia elongation and inhibiting Hh signals in chondrocytes [87]. This paradox suggests that more researches focused on the mechanisms of the interaction of primary cilia length and Hh signaling in OA are needed. Together, these studies indicated that Hh signaling is a potent therapeutic target for preventing or delaying cartilage degeneration.

### 5.2. Wnt Signaling Pathways

Primary cilia act as “antennae” of cells and are involved in the regulation of numerous signaling pathways, including Wnt [88]. Wnt signaling pathways are generally divided into canonical Wnt-*β*-catenin pathways and noncanonical Wnt-planar cell polarity (PCP) pathways. However, it is not clear what specific role primary cilia play in the Wnt signaling pathway. The first is that primary cilia negatively regulate canonical Wnt signaling. Elevated *β*-catenin levels are a hallmark of the activation of canonical Wnt signaling [89]. Lancaster et al. [90] reported stronger nuclear *β*-catenin staining in nonciliated cells compared to neighboring ciliated cells and that loss of cilia leads to enhanced Wnt signaling. Alternatively, Hassounah et al. [91] found that the presence of primary cilia was inversely associated with nuclear *β*-catenin in normal prostate tissue. This is consistent with the finding that primary cilia in cells inhibit canonical Wnt signaling. The second view is that primary cilia play a molecular switch between canonical and noncanonical Wnt signaling activity. Specifically, as in renal tubular epithelial cells, the presence of primary cilia can facilitate noncanonical Wnt signaling responses, while the loss of intact cilia enhances canonical Wnt signaling [92]. This seems to be an extension of the first view. The third opinion is that there is no relationship between cilia and Wnt signaling. For example, the IFT mutant mouse model did not change its Wnt activity compared to the wild-type [93]. These findings were validated in the zebrafish models. The maternal zygote ovule mutants that lack Ift88 lost all primary cilia, but still retained normal canonical and noncanonical Wnt signaling [94]. In recent years, apart from the signaling role of primary cilia, researchers have also argued about the role of Wnt signaling in ciliogenesis. Canonical Wnt signaling has been reported to induce primary ciliogenesis, while noncanonical Wnt signaling induced primary cilia disassembly [95]. In contrast, Bernatik et al. [96] blocked Wnt signaling by using the porcupine inhibitor LGK974, suggesting that Wnt ligand-mediated signaling is not required for primary ciliogenesis. The difference in these two conclusions can be attributed to differences in methodology, such as nutrient fluid, antibodies that mark the primary cilia, and experimental timing. Taken together, Wnt ligands may transmit signals in two cilia-dependent or independent ways.

Currently, research on cartilage-related pathways is mainly focused on canonical Wnt-*β*-catenin pathways. Canonical Wnt signaling pathways are mediated by *β*-catenin, which accumulates in the cytoplasm and then is transferred to the nucleus to form complexes with DNA-binding T-cell factors (TCFs), resulting in the activation of target gene transcription. The Wnt signaling network is active in adult cartilage and is involved in the regulation of differentiation, proliferation, and maturation during the life cycle of chondrocytes [97]. According to the human genetic experimental analysis, FRZB and DKK1, which are secreted inhibitor of Wnt, can inhibit the hypertrophic differentiation of articular cartilage and reduce the OA-like alterations [98, 99]. Thus, the activation of canonical Wnt signaling may play a detrimental role in OA development. Furthermore, increased levels of *β*-catenin and activation of the Wnt signal result in premature differentiation of chondrocytes and destruction of OA-like articular cartilage in Col2a1-CreER^T2^ and *β*-catenin^fx(Ex3)/wt^ transgenic mice [100]. It indicates that inhibition of Wnt signaling may be a therapeutic target to attenuate OA phenotypes. However, inhibition of *β*-catenin signal transduction in articular chondrocytes leads to increased chondrocyte apoptosis and articular cartilage destruction in Col2a1-ICAT transgenic mice [101]. Taken together, human and animal experiments provide evidence that canonical Wnt signaling is involved in OA pathogenesis and suggest that Wnt signaling appears to have a dual role in articular cartilage. There is evidence that a certain level of Wnt signaling is required to maintain the healthy state of articular cartilage [102]. How Wnt signals should be regulated in the future to restore articular cartilage homeostasis requires further study.

### 5.3. IL-1*β*-Related Signaling Pathways

In the development of OA, the role of inflammatory factors cannot be ignored. In inflammatory pathologies, the quintessentially proinflammatory cytokines interleukin-1*β* (IL-1*β*) and its receptors are upregulated as part of the broad spectrum of inflammatory mediators activated in many cell types. In chondrocytes, the IFT system in primary cilia is required for proinflammatory signaling of IL-1*β* [31]. Reportedly, IL-1*β* induces primary cilia elongation through protein kinases (including PKA) and NF*κ*B signaling pathways, which in turn increases nitric oxide and prostaglandin E2 release and promotes the occurrence of inflammatory reactions, while mutations in the IFT88 gene lead to cilia deletion in chondrocytes, resulting in a reduction in the inflammatory response [103–105]. Moreover, according to the research of Fu et al. [28], IL-1*β* (1 ng/ml) intervention in isolated chondrocytes increased the length of primary cilia to a median value of 2.21–2.84 *μ*m. However, this extension can be blocked by mechanical load, hypotonic challenge, and TRPV4 activation.

### 5.4. Hypoxia-Related Signaling Pathway

There is no capillary network in cartilage, so the chondrocyte microenvironment is usually in an anoxic state. Due to the hypoxic microenvironment of cartilage, hypoxia-inducible factors play an important role in cartilage development and homeostasis [106]. Among them, hypoxia-inducible factor-1 alpha (HIF-1*α*) is a protective factor for cartilage destruction, while HIF-2*α* is a catabolic factor in OA [107, 108]. It has been found that nuclear expression of both HIF-1*α* and HIF-2*α* was elevated under hypoxia stimulation, along with a decrease in the incidence of primary cilia [109]. This proves that the two may participate together in the regression of the primary cilia. Interestingly, knockout or overexpression HIF-1*α* or HIF-2*α* individually suggests that HIF-2*α* can induce a reduction in primary cilia and promote OA development, rather than HIF-1*α* [110]. This finding indicates that HIF-2*α* may be a new target for OA therapy. In addition, the presence of HIF-2*α* was found at the base of the chondrocyte primary cilia [33]. IL-1*β*-treated chondrocytes, with increased HIF-2*α* expression and gradual accumulation at the ciliary base and axoneme, further drive primary ciliary elongation and exacerbate inflammation.

### 5.5. Other Signaling Pathways

Studies have found the presence of multiple matrix receptors such as integrins (*α*2, *α*3, and *β*1) and NG2 on chondrocyte primary cilia, implicating that primary cilia participate in the signaling process associated with the synthesis and maintenance of the extracellular matrix [111]. The most typical receptor is integrin, a heterogeneous dimer transmembrane receptor that binds to many cartilage extracellular matrix molecules [112]. Within the cell, these receptors form complexes with associated ligands (including collagen, fibronectin, and laminin), activate the intracellular signal cascade, and regulate the gene expression of matrix molecules [113]. Therefore, primary cilia mutations that cause integrin signaling abnormalities may be the main cause of cartilage extracellular matrix degradation and one of the possible mechanisms of OA. In addition, GAL3, a key regulator of cartilage homeostasis and primary cilia formation in mice, is the base of chondrocyte primary cilia, whose absence promotes chondrocyte apoptosis through the mitochondrial pathway [114].

## 6. Conclusions and Perspectives

OA is a disabling joint disorder, and mechanical loading is an important pathogenesis [115]. A wide variety of cell types exhibit mechanosensitive behavior across cell activity, and there is growing evidence that primary cilia are key mechanosensory organelles in chondrocytes [29, 116]. In the articular cartilage, mechanical loading is essential for development, health, and homeostasis through the control of ECM synthesis and catabolism. It is therefore to be expected that primary cilia will have a significant impact not only on bone dysplasia but also on joint degenerative diseases, including OA [117–119].

Primary cilia are found in all murine chondrocytes and in 96% of equine chondrocytes, as well as in human and bovine chondrocytes [54, 56, 120]. Through transmission electron microscopy, electron tomography, and confocal microscopy, all cilia were straight in the absence of matrix, while various bending patterns were observed in chondrocyte cilia within a cartilage matrix or in situ [121, 122]. Thus, this observation suggests that, in addition to cilia lengthening, cilia bending is also a form of response to mechanical forces in the cartilage matrix. However, no research has been performed to clarify how the amplitude and frequency of cilia bending can affect OA diseases. Additionally, there are several key questions remain to be addressed, including but not limited to the morphological changes and specific signaling mechanisms of primary cilia-mediated OA, how to regulate primary cilia to repair articular cartilage, and the interaction between the chondrocyte primary cilia and the extracellular matrix. Regardless, targeting primary cilia may be a hot topic for future OA therapy.

## Figures and Tables

**Table 1:** The protein or signaling factors in the chondrocyte primary cilia.

Name	Definition	Location	Study related to cartilage development	Ref.
TRPV4	A nonselective cation channel	Ciliary membrane and axoneme	In this study, TRPV4 activation by mechanical, hypoosmotic, and pharmaceutical stimulation blocked IL-1*β*-mediated inflammatory signaling and destruction of the articular cartilage matrix by HDAC6-dependent modulation of ciliary tubulin.	[28]
PC1	Eleven transmembrane helix proteins	Cell membrane and cytoplasm	Wann et al. have demonstrated that the chondrocyte cilium is the downstream receptor of ATP-induced Ca^2+^ signaling and suggest that defective PC1 processing leads to disrupted signaling in Tg737^ORPK^ mutant cells.	[29]
PC2	Six transmembrane helices	Ciliary membrane	Thompson et al. have reported that upon mechanical stimulation, PC2 ciliary localization increases, which activates purinergic Ca^2+^ signaling, upregulates matrix gene expression, and protects cilia from mechanically induced disassembly.	[30]
HDAC6	a tubulin deacetylase	Axoneme	Fu et al. have shown that mechanical loading activates HDAC6 and disrupts tubulin acetylation and cilia elongation, which inhibits IL-1*β*-induced release of proinflammatory mediators, nitric oxide (NO), and prostaglandin E2 (PGE 2).	[31]
Arl13b	The membrane bound GTPase, a key regulator of the ciliary trafficking	Ciliary membrane and axoneme	Thorpe et al. have demonstrated that the prevalence and length of normalized primary cilia are dramatically reduced and Arl13b expression at the distal tip is increasing in AKU chondrocytes, which manifests itself as articular cartilage degeneration, resulting in inhibition of ligand-induced hedgehog signaling.	[32]
HIF-2*α*	a DNA-binding transcription factor	Ciliary base	This study indicates that the primary cilium regulates HIF signaling during inflammation.	[33]
TGF-*β*R	The orphan G-protein-coupled receptor	Ciliary pocket	Kawasaki et al. have reported that TGF-*β* suppresses Ift88 expression and reduces the length of primary cilia in chondrocytic ATDC5 cells, which may be the mechanisms of cartilage pathophysiology.	[34]
Gpr161	The orphan G-protein-coupled receptor	Primary cilia	Hwang et al. have reported that Gpr161 regulates limb patterning, endochondral, and intramembranous skeletal morphogenesis in a cilium-dependent way.	[35]

PC1: polyc ystin-1; PC2: polycystin-2; HDAC6: histone deacetylase 6; Arl13b: ADP-ribosylation factor-like protein 13B; HIF-2*α*: hypoxia-inducible factor 2 alpha.
